# Regulation of denitrification at the cellular level: a clue to the understanding of N_2_O emissions from soils

**DOI:** 10.1098/rstb.2011.0321

**Published:** 2012-05-05

**Authors:** Lars R. Bakken, Linda Bergaust, Binbin Liu, Åsa Frostegård

**Affiliations:** 1 Department of Plant and Environmental Sciences, Norwegian University of Life Sciences, PO Box 5003, 1432 Aas, Norway; 2 Department of Chemistry, Biotechnology and Food Sciences, Norwegian University of Life Sciences, PO Box 5003, 1432 Aas, Norway

**Keywords:** nitrous oxide, soil, physiology, regulation, mitigation

## Abstract

Denitrifying prokaryotes use NO_x_ as terminal electron acceptors in response to oxygen depletion. The process emits a mixture of NO, N_2_O and N_2_, depending on the relative activity of the enzymes catalysing the stepwise reduction of NO_3_^−^ to N_2_O and finally to N_2_. Cultured denitrifying prokaryotes show characteristic transient accumulation of NO_2_^−^, NO and N_2_O during transition from oxic to anoxic respiration, when tested under standardized conditions, but this character appears unrelated to phylogeny. Thus, although the denitrifying community of soils may differ in their propensity to emit N_2_O, it may be difficult to predict such characteristics by analysis of the community composition. A common feature of strains tested in our laboratory is that the relative amounts of N_2_O produced (N_2_O/(N_2_+N_2_O) product ratio) is correlated with acidity, apparently owing to interference with the assembly of the enzyme N_2_O reductase. The same phenomenon was demonstrated for soils and microbial communities extracted from soils. Liming could be a way to reduce N_2_O emissions, but needs verification by field experiments. More sophisticated ways to reduce emissions may emerge in the future as we learn more about the regulation of denitrification at the cellular level.

## 1. Denitrification in soils: a major source of atmospheric N_2_O

Human activities have more than doubled the annual input of reactive nitrogen to the biosphere compared with prehistoric rates. This anthropogenic reactive N is primarily fertilizer N, biologically fixed N (in legume crops) and NO_x_ from combustion. The ongoing amplification of the global N cycle has already altered the biogeochemical processes of natural ecosystems, their trophic dynamics and biological diversity [1]. The ultimate fate of anthropogenic nitrogen is a return to the atmosphere, either as N_2_, N_2_O or NO, which are the gaseous products of microbial red/ox-transformations of mineral nitrogen. The N-gas product-stoichiometry of these processes is controlled by the ecology and regulatory biology of the organisms involved, as modulated by environmental factors. A better understanding and quantification of these processes is urgently needed to improve our chances to minimize N_2_O emissions [2]. The current accumulation of N_2_O in the atmosphere accounts for approximately 9 per cent of estimated anthropogenic global warming and is recognized as the major factor causing destruction of stratospheric ozone [3].

The global N_2_O emissions from cultivated soils have been estimated at 3.3 Tg N_2_O yr^−1^ [4], which is around 50 per cent of the estimated global anthropogenic N_2_O emission. A substantial part of the remaining anthropogenic N_2_O emissions (from water bodies and uncultivated areas) are driven by nitrogen losses from agroecosystems as well [5]. There is thus little doubt that terrestrial habitats (both cultivated and uncultivated) are one of the most important sources of atmospheric N_2_O. Denitrification is probably a much more potent N_2_O source than nitrification, as indicated by the low N_2_O/NO_3_^−^ product stoichiometry of nitrification [6]. Tracer studies suggesting a major role of nitrification may possibly have confounded coupled nitrification–denitrification via nitrite [7].

Few tools are available to reduce the emissions from natural ecosystems, apart from reducing the inputs of anthropogenic reactive nitrogen. The effect of such reductions may be slow, however, owing to the long residence time of reactive nitrogen (as biomass and soil organic N) within the systems [8]. Cultivated areas, on the other hand, are intensively managed by drainage, cropping, tillage, liming, fertilization and use of agrochemicals. These operations should hold a potential for reducing the N_2_O emissions, provided that we know how they affect the activity of the organisms responsible for N_2_O production in the soil. Despite several decades of research on N_2_O emissions, however, few (if any) practical ways to achieve substantial reductions of N_2_O emissions have been invented.

Why is there so little progress? How can we improve our efforts? We suspect that one of the reasons for the current shortcoming of the research on N_2_O emission is that there has been too little cross-talk between disciplines. Great progress has been achieved from studies of the biochemistry, regulatory biology and physiology of denitrifying prokaryotes over the last two decades, driven by molecular biology. Yet, too little of this knowledge has reached the agronomists/biogeochemists/earth scientists who have made great progress within their own fields. Most experiments within each of the disciplines mentioned are designed to solve ‘disciplinary’ problems, by scientists with only rudimentary understanding of how adjacent disciplines think and work, and their recent progress. We believe that better interaction and discussions between the disciplines hold a potential for progress in the search for ways to mitigate N_2_O emission.

The present paper is an attempt to illustrate this potential, by reviewing recent progress in a series of experimental approaches dedicated to understanding the mechanisms at the cellular level which control the N_2_O/(N_2_+N_2_O) product ratio of denitrification. This ratio is of utmost importance for the earth sciences in their attempt to link N_2_O emissions to the current amplification of the nitrogen cycle. Before diving into the microbiological investigations, we need to briefly recapitulate some research on N_2_O emissions within the earth sciences.

## 2. Field experiments

N_2_O emissions from agricultural soils have been studied intensively in numerous field trials on all continents, reviewed and analysed thoroughly by Stehfest & Bouwman [4]. Excess fertilizer N doses result in high emissions, but N_2_O emissions correlate only poorly with the fertilizer N level when within realistic ranges. Numerous other factors are involved in controlling the N_2_O emission from soils, which contribute to the huge spatio-temporal variation in emissions in field trials [4,9]. An unfortunate consequence of the spatio-temporal variation is that the power of statistical tests of such experiments is low, i.e. although the experimental treatments may have a real effect, there is a high risk of erroneously concluding that there was no effect (type II error). A number of management effects may have been erroneously rejected for this reason. A simple calculation of the confidence interval of the difference between contrasting treatments could reveal the pitfall; i.e. the substantial effect of a management could have existed without being detected as statistically significant. This is often neglected, possibly because it is frustrating to illuminate the low statistical power of the experiment.

Another problem with field trials is that the observations tend to be ‘anecdotal’ because the effects of specific operations on N_2_O emissions are likely to be conditional, i.e. dependent on several other variables such as soil type, climate and other management operations. Drainage of the soils is a good example: drainage of paddy rice soils (drainage is often included in the annual management of such fields) will enhance the N_2_O emission [10]; in farmed organic soils, the lowering of the water table (i.e. drainage) may or may not reduce N_2_O emissions depending on other factors [11], whereas adequate artificial drainage to sustain a low water table depth throughout the whole year appears to reduce N_2_O emissions from grassland soils [12]. Another example is tillage, which may result in higher or lower annual N_2_O emissions, apparently depending on the climate and possibly a number of soil variables ([13] and references therein).

A third shortcoming of nearly all field experiments is that the N_2_O/N_2_ product ratio has not been measured, primarily because it has proved difficult to obtain reliable field measurements of N_2_ production [14]. The acetylene inhibition method may be used, but is likely to underestimate denitrification owing to diffusion limitation [15] and acetylene-catalyzed oxidation of NO [16]. Tracer-based measurements are possible [17], but have not been used much because they are complicated, demanding continuous monitoring of both gas emissions and the tracer (^15^N) concentration in the nitrate/nitrite pool of the soil. The scarcity of reliable measurements of N_2_ production in field experiments is most unfortunate, since the N_2_O/N_2_ product ratios should be a target for designing mitigation options [2].

The potential benefit of measuring both N_2_ and N_2_O production is illustrated by two studies of intact soils from field experiments conducted by Zaman *et al*. [18,19], where they quantified both N_2_O and N_2_ production (the latter by the acetylene inhibition technique). They found that liming invariably reduced the N_2_O/N_2_ product ratio, and the effect on net N_2_O emission was either nil [18] or positive (higher emission from limed soil [19]). Their quantification of N_2_ production by the acetylene inhibition method may have been biased for the reason mentioned earlier (lack of steady-state conditions owing to restricted diffusion, and NO scavenging by acetylene). Nevertheless, their observation of an altered N_2_O/N_2_ product ratio is probably valid since we may assume that the bias is the same in all treatments.

## 3. Ecosystem modelling

Biogeochemical modelling of denitrification and its N_2_O production are based on relatively crude models of the process and its regulation at the cellular level. Much effort has been invested in modelling the heat-, water- and not the least oxygen-transport within the soil matrix [20]. Less effort has been invested in refining the modelling of denitrifying community phenotype, which is a ‘black box’ in most models, based on relatively antique parameters for enzyme and growth kinetics. In addition, all soils are assumed to harbour the same community phenotype [9,21]. It appears bluntly wrong to assume that all community phenotypes are equal, as judged from investigations of community phenotypes of contrasting soils [22,23]. Despite these (and other) gross simplifications, the simulated annual N_2_O emissions are often in reasonable agreement with measured values for contrasting soils [24] and for contrasting soil management scenarios [25], although the temporal variation is still a challenge [20,24].

Simplifications are necessary in biogeochemical ecosystem modelling, but it seems likely that the existing models could be improved through a dialogue between the modellers and the research community working with the genetic and physiological basis of denitrification. This is particularly true if the purpose of the modelling is to design ‘mitigation measures’, i.e. soil and fertilization routines that reduce the N_2_O emissions. As stated previously, a reduction in the N_2_O/(N_2_ + N_2_O) product ratio of denitrification should be a target when searching for mitigation measures. The existing models are possibly inadequate for this purpose, although this is unknown because we lack reliable data for N_2_ production in field experiments to test their predictions. It would be arrogant (but possibly legitimate) to claim that when a nearly perfect match is found between measured and simulated annual N_2_O emissions, the biogeochemical model is right for a number of wrong reasons. It is less arrogant (but fully legitimate) to claim that if used to simulate phenomena for which the models are not rigorously tested, their predictions are rather hypothetical.

Our impression is that field experiments and modelling of N_2_O emissions have not made much progress regarding mitigation of N_2_O emissions. This is reflected in several reviews of available mitigation options, which generally recommend operations that (i) secure synchronization of mineralization with the need for mineral N by growing crops and (ii) minimize the mineral N concentrations in the soil during off-season [26–31], which are no more than the general recommendations for good agronomic practice to minimize nitrate leaching [32]. A perpetuation of field experiments and modelling of N_2_O emissions along the same track will probably not result in substantial progress regarding mitigations. That is not to say that such activities should be terminated. Field emission data are extremely important for several obvious reasons, and any hypothetical mitigation must be tested in field experiments. We are convinced that if there exist novel ways to reduce N_2_O emissions, such mitigation options are more likely to be found through studies of the ecology and regulatory biology of denitrifying prokaryotes, with a deliberate focus on their ability to express N_2_O reductase. We have dedicated ourselves to work along these lines, and the rest of this paper is a first attempt to summarize some of our findings.

## 4. N_2_O from denitrification depends on regulation at the cellular level

Denitrification is the stepwise reduction of NO_3_^−^ through NO_2_^−^, NO, N_2_O to N_2_, driven by four reductase enzymes NAR/NAP, NIR, NOR and N_2_OR, respectively. This enables the organisms to sustain respiratory metabolism during oxygen limitation, with NO_x_ as terminal electron acceptors. The denitrification proteome (NAR, NIR, NOR and N_2_OR plus several other proteins) is synthesized in response to oxygen depletion, and is blocked by high oxygen concentrations (both transcriptional and post-transcriptional control [33]).

**Figure 1.**
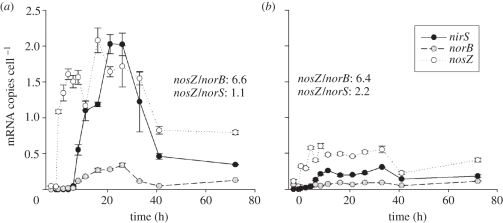
Transcription of *nirS*, *norB* and *nosZ* in *Paracoccus denitrificans* in response to oxygen depletion when grown in Sistrom's medium with 34 mM succinate as the main C source. Results are shown for two pH levels ((*a*) pH 7; (*b*) pH 6). The relative transcription of *nosZ* is expressed by *nosZ/norB* and *nosZ/nirS* ratios in each panel, based on the four highest transcript numbers recorded for each gene (from Bergaust *et al*. [37]).

The transcription of the genes coding for the individual reductases is controlled by a network of transcriptional regulators and ancillary factors [34,35] which respond to intra- and extracellular signals among which are oxygen and N-oxides (NO and NO_2_^−^). Although important ‘nuts and bolts’ of this regulatory network have been unravelled for a few model strains, such as *Paracoccus denitrificans*, much remains to be discovered before we are able to fully understand the phenotypic response. For instance, we have found that phenotypic responses of various mutants of *P. denitrificans* suggest that the transcription of *nar* requires *both FnrP* and *NarR* (dual control), while the transcription of *nosZ* (coding for N_2_OR) is equally effective with only *FnrP* (in response to oxygen depletion) or *NNR* (responding to NO) [36]. Another conspicuous finding regarding the regulation in *P. denitrificans* is that N_2_OR is expressed much earlier than NIR and NOR (and possibly NAR), as illustrated in figure 1. Moreover, only a fraction of the cells are actually able to express NIR and NOR in due time before all the oxygen has been depleted [37,38]. We hypothesize that this is due to a stochastic initiation of the transcription of *nir* and *nor*, which then accelerates owing to product stimulation (NO accelerates transcription of both genes). In contrast, nearly 100 per cent of the cells appear to express N_2_OR, as judged from the specific rate of reduction of externally supplied N_2_O [37,38].

**Figure 2.**
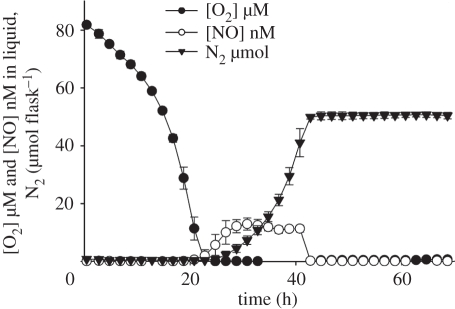
The typical denitrification phenotype of *P. denitrificans* in response to oxygen depletion in a 50 ml batch of Sistrom's medium with 2 mM NO_3_^−^ and pH = 7. The graph shows O_2_ and NO concentration in the liquid (as µM and nM, respectively) and cumulated N_2_ production (as µmol flask^−1^) . NO concentrations are sustained around 15 nM (i.e. 20 nmol flask^−1^) during the period of active denitrification (which lasts until all the nitrate has been depleted and recovered as N_2_). The transient accumulation of N_2_O is below the detection limit of the system which is approximately 2 nmol flask^−1^ . Selected data from Bergaust *et al*. [37].

The peculiar patterns of regulation in *P. denitrificans* explains why this organism emits only traces of N_2_O (less than 2 nmol flask^−1^) during transition from oxic to anoxic conditions (figure 2).

**Figure 3.**
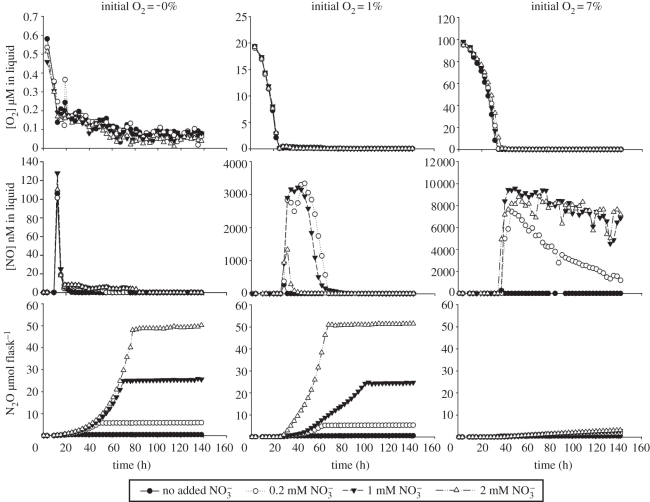
Denitrification phenotype of *Agrobacterium tumefaciens*, conditions similar to that for *P. denitrificans* (figure 2), but at three different initial concentrations of oxygen (indicated at top) and four concentrations of nitrate. The treatment ‘No added NO_3_^−^’ contains 17 µM NO_3_^−^as a part of the basal Sistrom's medium. *Agrobacterium tumefaciens* lacks N_2_O reductase, hence N_2_O is the end product. At initially low oxygen concentrations (∼0 v/v %), the cell density is low, hence depletion of oxygen is slow, and the organisms are able to perform a reasonably balanced transition from oxic to anoxic denitrification (marginal amounts of NO accumulates transiently). At high initial oxygen concentrations (7 v/v %), oxic growth secures high cell density prior to oxygen depletion, hence final depletion of O_2_ occurs very fast, resulting in unbalanced denitrification and paralysingly high NO concetrations. The figure is adapted from Linda Bergaust, PhD thesis 2007, Norwegian University of Life Sciences. The figure is a corrected version of fig. 2 in Bergaust *et al.* [39], which had seriously underestimated NO concentrations (the Journal refused to accept this as an erratum).

The denitrification phenotype of *P. denitrificans* at pH 7 demonstrates an outstanding performance regarding efficient reduction of NO_x_ all the way to N_2_, with only minor emissions of both NO and N_2_O (less than 2 nmol N_2_O flask, or less than 0.004% of all NO_3_-N reduced to N_2_). If the denitrifying communities of soils performed equally well, their contribution to emission of NO and N_2_O would be negligible. The performance of *P. denitrificans* appears to be exceptional, however. A strain that has been studied in equal detail in our laboratory is the soil bacterium *Agrobacterium tumefaciens* [39]. This organism is unable to reduce N_2_O to N_2_ because it lacks *nosZ*, i.e. the gene coding for N_2_OR. Strains which lack *nosZ* occur within many genera of denitrifying prokaryotes, and if organisms with such truncated denitrification apparatus were dominating in soils, it would obviously result in high N_2_O/(N_2_+N_2_O) product ratios of denitrification. But *A. tumefaciens* contrasts with *P. denitrificans* in another conspicuous way: when confronted with a rapid transition from oxic to anoxic conditions, it appears unable to perform a balanced expression of NIR and NOR, resulting in extremely high emissions of NO, as illustrated in figure 3.

**Figure 4.**
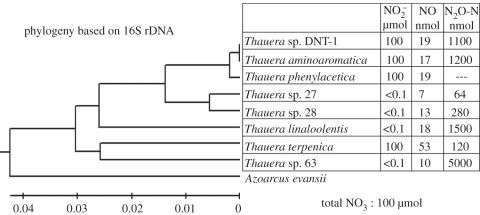
Relationship between phylogeny and the transient accumulation of NO_2_^−^, NO and N_2_O for different strains of *Thauera*; experimental conditions were similar to that used for *P. denitrificans* (figure 2). The phylogenetic tree was constructed using UPGMA method (for details see Liu *et al.* [40]), and the table shows the maximum amounts of the three intermediates transiently accumulated: NO_2_^−^ (µmol flask^−1^), NO and N_2_O-N (nmol flask^−1^) during batch incubations of 50 ml culture (2 mM NO_3_^−^) in 120 ml reaction vessels. 100 µmol NO_2_^−^ implies that all NO_3_^−^ accumulated as NO_2_^−^ during the first phase of denitrification. To convert nmol NO to nM in the liquid: 1 nmol flask^−1^ is equivalent to 0.7 nM in the liquid. *Thauera Phenylacetica* lacked *nosZ*, and converted all nitrate to N_2_O. Data assembled from Liu *et al.* [40].

We have also analysed the denitrification regulatory phenotype (DRP) of a number of reference strains and recently isolated strains within the genus *Thauera* under nearly identical experimental conditions as that for *P. denitrificans* shown in figure 2 [40]. One of the strains lacked *nosZ* and produced 100 per cent N_2_O, while the seven other strains had all the necessary genes to make a complete denitrification from NO_3_^−^ to N_2_. A common feature of all the *Thauera* strains was robust control of NO at nanomolar levels, very similar to *P. denitrificans* (5–35 nM in the liquid phase). They deviated grossly, however, regarding the transient accumulation of both NO_2_^−^ and N_2_O. Some of the strains reduced all NO_3_^−^ to NO_2_^−^ before expressing NIR and NOR, wheras others accumulated negligible amounts of NO_2_^−^. The strains were also different regarding the transient accumulation of N_2_O, ranging from 0.06 to 5 per cent of all NO_3_^−^-N finally reduced to N_2_. The results are summarized in figure 4, together with their phylogenetic relationship based on 16S rDNA.

There exists a plethora of similar studies where the transient accumulation of N_2_O has been studied in a similar way for complex communities [41,42], biofilms [43] and in cultured denitrifiers [44–54]. It is difficult to summarize these results in a consistent way, since the experimental conditions are widely different from one experiment to the other. But the general impression is that the denitrification phenotypes of prokaryotes are profoundly different regarding their ‘intrinsic’ propensity of emitting N_2_O, as assessed by the amounts of N_2_O accumulating transiently when switching from oxic to anoxic respiration. These differences between denitrification phenotypes are most probably owing to differences in the regulatory network controlling the transcription of their denitrification genes (and possibly post-translational regulation). Thus, we propose a term ‘DRP’ [38], which is a set of variables that characterize the organisms ability to perform a balanced and effective transition from oxic to anoxic respiration with marginal emissions of intermediates.

Assuming that denitrifying bacteria are profoundly different regarding their DRPs, a crucial question is whether these characteristics are in any way related to phylogeny of the organisms. If such relationships exist, the analysis of denitrification community DNA would possibly enable us to predict characteristics of the community phenotype, although the task would be far from trivial owing to interactions via the intermediates NO_2_^−^ and NO. Further on, the phylogeny of the DRP could also shed some light on the evolution of the various DRP traits and their fitness value.

However, we have scant knowledge about the phylogeny of various DRP traits, since this has hardly been studied at all in the past. The results already mentioned for the closely related strains within *Thauera* [40] demonstrated surprisingly different DRPs among such closely related strains, both regarding their transient accumulation of NO_2_^−^ and N_2_O, but not for NO. The allocation of these DRPs within the phylogenetic tree does not suggest any systematic relationship between phylogeny and the various DRP traits.

To sum up, it seems clear that denitrification phenotypes are profoundly different regarding their propensity to emit N_2_O, assessed by their transient accumulation of N_2_O when initiating denitrification in response to oxygen depletion. They are also very different regarding their transient accumulation of nitrite (NO_2_^−^). We have too few rigorous comparative investigations to draw conclusions on the possible relationship between these phenotypes and taxonomy/phylogeny, but the study of closely related strains of *Thauera* do indicate that we may expect to find as much variation within taxonomic groups as between them. This would mean that characterizing the composition of denitrifying communities by metagenomic sequencing of genes coding for 16S rRNA or functional denitrification genes has limited value as a predictor of the communities' propensity to emit N_2_O. It would not imply, however, that no relationship will be found between the community composition and its propensity to emit N_2_O. Attempts have been made, with variable success, in ecophysiological studies of N_2_O formation [55–58]. Although such approaches cannot be expected to reveal causal relationships between genetic composition and phenotype of soil microbial communities, they may serve as hypothesis-generating observations. In order to find causal relationships, we may have to dive deeper than to phylogeny, possibly by identifying regulatory gene sequences that characterize various DRPs across phyla.

## 5. Denitrification is affected by pH

A recurring observation in ecological studies of denitrification has been that the product ratio of denitrification is strongly affected by pH [59,60]. Soils have inherently different pH depending on their parent mineral material modulated by biological processes, and the N_2_O/(N_2_+N_2_O) product ratio appears to be negatively correlated with pH within the normal range for agricultural soils (pH 5–8). This would indicate that liming slightly acidic soils could be a way to reduce N_2_O emissions, but this has been effectively ignored until recently. Why did it take so long time to discover this? Possibly for two reasons. One is that the immediate response to liming is a drastic increase in N_2_O emission [61], most probably because it causes a transient enhancement of net N mineralization and nitrification. A second reason may be that the mechanism causing the observed pH effect on the N_2_O/N_2_ product ratio of denitrification was not well understood.

How can the pH affect the N_2_O/N_2_ product ratio of denitrification? Our studies of the model strain *P. denitrificans* [37] shed some light on this:

— At pH 7, *P. denitrificans* emits nearly no N_2_O when switching from oxic to anoxic denitrification in batch cultures.
— Lowering the pH of the medium resulted in increasing transient accumulation of N_2_O, and at pH 6, it produces nearly 100 per cent N_2_O (no N_2_O reductase activity).
— The lack of N_2_O reductase activity at pH 6 was *not* caused by low relative transcription rate of *nosZ* compared with that of the other denitrification genes; the ratio between mRNA copy numbers for *nosZ* and that for *nirS* was practically unaffected by pH (figure 1).
— Neither is it due to a particularly narrow pH range for the activity of the N_2_O reductase enzyme (compared with that of the other denitrification enzymes): N_2_O reductase expressed at pH 7 was functioning well at pH 6 when tested *in vivo*.

Our tentative conclusion is that low pH hinders the synthesis of a functional N_2_O reductase enzyme, most probably by interfering with the assembly of the enzyme in the periplasm (which is the location of the functional enzyme).

The knee-jerk reaction of many microbial ecologists to this is that *P. denitrificans* is no more than a model strain with little relevance to natural habitats. However, we have collected convincing evidence that the phenomenon occurs in soils:

— Soils from long-term liming experiments demonstrate a pervasive control of pH on the (N_2_O/N_2_ + N_2_O) product ratio, although the relative amount of *nosZ* genes and their transcripts (compared with that of the other denitrification genes) appears unaffected by pH within the range 5–8 [62].
— Experiments with cells extracted from the same soils (long-term liming experiments) show essentially the same phenomenon for cells from neutral soils, but cells from acid soils appear unable to produce functional N_2_O reductase even when transferred to neutral conditions [40].
— Experiments with cells extracted from organic soils with different cultivation history, pH and N_2_O emissions [63], demonstrated the same pH dependency, although the contrasting community composition appeared to modulate the pH sensitivity [23,58].
— All strains (Gram positives and Gram negatives) tested in our laboratory so far have shown the same pH dependency of the relative activity.
— Ongoing investigations of soils sampled from long-term field experiments (Austria, Hungary, Nepal and China) demonstrate invariably that the (N_2_O/N_2_ + N_2_O) product ratio of denitrification under anoxic incubation of intact soils is negatively correlated with pH, independent of the mechanism controlling the soil pH (liming, parent material or excess fertilization).

## 6. Concluding remarks

Our investigations demonstrate that the N_2_O/(N_2_+N_2_O) product ratio of denitrification is pervasively controlled by pH, be it in pure cultures of denitrifying bacteria or in soils. Our in-depth studies of the model strain *P. denitrificans* showed that the effect is primarily owing to interference with the assembly of the enzyme, rather than a narrow pH range for the N_2_O reductase enzyme. The recurring observations of similar pH effect on denitrification in soils indicate that it is a general phenomenon in nature. The findings suggest that the ongoing soil acidification of agricultural soils by intensification of agriculture and excessive use of N-fertilizers, as convincingly demonstrated for China [64], may enhance the N_2_O emissions drastically. We hypothesize that careful adjustment of pH in agricultural soils will reduce N_2_O emissions from slightly acidic soils. This needs to be tested rigorously in field trials, however. More sophisticated ways to reduce N_2_O emissions may emerge in the future as we learn more about the ecology and the regulatory biology of denitrifying prokaryotes.

## References

[1] Elser J. J. Andersen T. Baron J. S. Bergstron A. K. Jansson M. Kyle M. Nydick K. R. Steger L. Hessen D. O. 2009 Shifts in Lake N:P stoichiometry and nutrient limitation driven by atmospheric nitrogen deposition. Science 326, 835–837 10.1126/science.1176199 (doi:10.1126/science.1176199) 19892979

[2] Schlesinger W. J. 2009 On the fate of anthropogenic N. Proc. Natl Acad. Sci. USA 106, 203–208 10.1073/pnas.0810193105 (doi:10.1073/pnas.0810193105) 19118195 PMC2613040

[3] Ravishankara A. R. Daniel J. S. Portmann R. W. 2009 Nitrous oxide (N_2_O): the dominant ozone-depleting substance emitted in the 21st century. Science 326, 123–125 10.1126/science.1176985 (doi:10.1126/science.1176985) 19713491

[4] Stehfest E. Bouwman L. 2006 N_2_O and NO emission from agricultural fields and soils under natural vegetation: summarizing available measurement data and modelling of annual emissions. Nutrient Cycl. Agroecosyst. 74, 207–228 10.1007/s10705-006-9000-7 (doi:10.1007/s10705-006-9000-7)

[5] Galloway J. N. 2004 Nitrogen cycles: past, present and future. Biogeochemistry 70, 153–226 10.1007/s10533-004-0370-0 (doi:10.1007/s10533-004-0370-0)

[6] Mørkved P. T. Dörsch P. Bakken L. R. 2007 The product ratio of nitrification and its dependence on long term changes in pH. Soil Biol. Biochem. 39, 2048–2057 10.1016/j.soilbio.2007.03.006 (doi:10.1016/j.soilbio.2007.03.006)

[7] Russow R. Stange C. F. Neue H. U. 2009 Role of nitrite and nitric oxide in the process of nitrification and denitrification in soil: results from 15N tracer experiments. Soil Biol. Biochem. 41, 785–795 10.1016/j.soilbio.2009.01.017 (doi:10.1016/j.soilbio.2009.01.017)

[8] Bakken L. R. Bleken M. A. 1998 Temporal aspects of N-enrichment and emission of N2O to the atmosphere. Nutrient Cycl. Agroecosystems 52, 107–121 10.1023/A:1009760623188 (doi:10.1023/A:1009760623188)

[9] Bakken L. R. Dörsch P. 2007 Nitrous oxide emissions and global changes: modelling approaches. In Biology of the nitrogen cycle, Ch. 25 (eds Bothe H. Ferguson S. J. Newton W. E.), pp. 382–395 Amsterdam, The Netherlands: Elsevier

[10] Wang J. Y. Jia J. X. Xiong Z. Q. Khalil M. A. K. Xing G. X. 2011 Water regime–nitrogen fertilizer–straw incorporation interaction: field study of nitrous oxide emissions from a rice agroecosystem in Nanjing, China. Agric. Ecocyst. Environ. 141, 437–446 10.1016/j.agee.2011.04.009 (doi:10.1016/j.agee.2011.04.009)

[11] Berglund O. Berglund K. 2011 Influence of water table level and soil properties of greenhouse gases from cultivated peat soil. Soil Biol. Biochem. 43, 923–931 10.1016/j.soilbio.2011.01.002 (doi:10.1016/j.soilbio.2011.01.002)

[12] Dobbie K. E. Smith K. A. 2006 The effect of water table depth on emissions of N_2_O from a grassland soil. Soil Use Manage. 22, 22–28 10.1111/j.1475-2743.2006.00002.x (doi:10.1111/j.1475-2743.2006.00002.x)

[13] Rochette P. 2008 Estimation of N2O emissions from agricultural soils in Canada. 1. Development of a country-specific methodology. Can. J. Soil Sci. 88, 641–654

[14] Groffman P. M. 2006 Methods for measuring denitrification: diverse approaches to a difficult problem. Ecol. Appl. 16, 2091–2122 10.1890/1051-0761(2006)016[2091:MFMDDA]2.0.CO;2 (doi:10.1890/1051-0761(2006)016[2091:MFMDDA]2.0.CO;2) 17205891

[15] Kliewer B. A. Gilliam J. W. 1995 Water table management effects on denitrification and nitrous-oxide evolution. Soil Sci. Soc. Am. J. 59, 1694–1701 10.2136/sssaj1995.03615995005900060027x (doi:10.2136/sssaj1995.03615995005900060027x)

[16] Murray R. E. Knowles R. 2003 Production of NO and N_2_O in the presence and absence of C_2_H_2_ by soil slurries and batch cultures of denitrifying bacteria. Soil Biol. Biochem. 35, 1115–1122 10.1016/S0038-0717(03)00163-9 (doi:10.1016/S0038-0717(03)00163-9)

[17] Bergsma T. T. Ostrom N. E. Emmons M. Roberstson G. P. 2001 Measuring simultaneous fluxes of N_2_O and N_2_ in the field using the ^15^N-gas ‘nonequilibrium’ technique. Environ. Sci. Technol. 35, 4307–4312 10.1021/es010885u (doi:10.1021/es010885u) 11718347

[18] Zaman M. Nguyen M. L. Matheson F. E. Blennerhassett J. D. Quin B. F. 2007 Can soil amendments (zeolite or lime) shift the balance between nitrous oxide and dinitrogen emissions from pasture and wetland soils receiving urine or urea-N? Aust. J. Soil Res. 45, 543–553 10.1071/SR07034 (doi:10.1071/SR07034)

[19] Zaman M. Nguyen M. L. Saggar S. 2008 N_2_O emission from pasture and wetland soils with and without amendments of lime and zeolites under laboratory conditions. Aust. J. Soil Sci. 46, 526–534 10.1071/SR07218 (doi:10.1071/SR07218)

[20] Schurgers G. Dörsch P. Bakken L. Leffelaar P. Haugen L. E. 2006 Modelling soil anaerobiosis from water retention characteristics and soil respiration. Soil Biol. Biochem. 38, 2637–2644 10.1016/j.soilbio.2006.04.016 (doi:10.1016/j.soilbio.2006.04.016)

[21] Chen D. Li Y. Grace P. Mosier A. R. 2008 N2O emissions from agricultural lands: a synthesis of simulation approaches. Plant Soil 309, 169–189 10.1007/s11104-008-9634-0 (doi:10.1007/s11104-008-9634-0)

[22] Cavigelli M. A. Robertson G. P. 2000 The functional significance of the denitrifier community composition in a terrestrial ecosystem. Ecology 81, 1402–1414 10.1890/0012-9658(2000)081 (doi:10.1890/0012-9658(2000)081)

[23] Dörsch P. Braker G. Bakken L. R. 2012 Community specific pH response of denitrification: experiments with cells extracted from organic soils. FEMS Mirobiol. Ecol. 79, 530–541 10.1111/j.1574-6941.2011.01233.x (doi:10.1111/j.1574-6941.2011.01233.x) 22093000

[24] Li C. S. Frolking S. Butterbach-Bahl K. 2005 Carbon sequestration in arable soils is likely to increase nitrous oxide emissions, offsetting reductions of climate radiative forcing. Clim. Change 72, 321–338 10.1007/s10584-005-6791-5 (doi:10.1007/s10584-005-6791-5)

[25] Frolking S. E. 1998 Comparison of N2O emissions from soils at three temperate agricultural sites: simulations of year-round measurements by four models. Nutr. Cycl. Agroecosys. 52, 77–105 10.1023/A:1009780109748 (doi:10.1023/A:1009780109748)

[26] Hillier J. Walter C. Malin D. Garcia-Suarez T. Mila-i-Canals L. Smith P. 2001 A farm-focused calculator for emission from crop and livestock production. Environ. Model. Softw. 26, 1070–1078 10.1016/j.envsoft.2011.03.014 (doi:10.1016/j.envsoft.2011.03.014)

[27] Insam H. Wett B. 2008 Control of GHG emission at the microbial community level. Waste Manage. 28, 699–706 10.1016/j.wasman.2007.09.036 (doi:10.1016/j.wasman.2007.09.036) 18053703

[28] Luo J. de Klein C. A. M. Ledgard S. F. Saggar S. 2010 Management options to reduce nitrous oxide emissions from intensively grazed pastures: a review. Agric. Ecosyst. Environ. 136, 282–291 10.1016/j.agee.2009.12.003 (doi:10.1016/j.agee.2009.12.003)

[29] Liebig M. A. Morgan J. A. Reeder J. A. Ellert B. H. Gollany H. T. Schuman G. E. 2005 Greenhouse gas contributions and mitigation potential of agricultural practices in northwestern USA and Western Canada. Soil Tillage Res. 83, 25–52 10.1016/j.still.2005.02.008 (doi:10.1016/j.still.2005.02.008)

[30] Johnson J. M. F. Reicosky D. C. Allmaras R. R. Sauer T.J. Venterea R. T. Dell C. J. 2005 Greenhouse gas contributions and mitigation potential of agriculture in the central USA. Soil Tillage Res. 83, 73–94 10.1016/j.still.2005.02.010 (doi:10.1016/j.still.2005.02.010)

[31] Albrecht A. Kandij S. T. 2003 Carbon sequestration in tropical agroforestry systems. Agric. Ecosyst. Environ. 99, 15–27 10.1016/S0167-8809(03)00138-5 (doi:10.1016/S0167-8809(03)00138-5)

[32] Granli T. Bøckman O. C. 1994 Nitrous oxide from agriculture. Norwegian J. Agric. Sci. Suppl. 12, 1–128

[33] Van Spanning R. J. M. Richardson D. Ferguson S. 2007 Introduction to the biochemistry and molecular biology of denitrification. In Biology of the nitrogen cycle*, Ch. 1* (eds Bothe H. Ferguson S. J. Newton W. E.), pp. 382–395 Amsterdam, The Netherlands: Elsevier

[34] Zumft W. 1997 Cell biology and molecular basis of denitrification. Microbiol. Mol. Biol. Rev. 61, 533–616 9409151 10.1128/mmbr.61.4.533-616.1997 PMC232623

[35] Zumft W. Kroneck P. M. H. 2007 Respiratory transformation of nitrous oxide (N2O) to dinitrogen by Bacteria and Archaea. Adv. Microbial. Physiol. 52, 109–197 10.1016/S0065-2911(06)52003-X 17027372

[36] Bergaust L. vanSpanning R. Frostegård Å. Bakken L. R. In press Expression of nitrous oxide reductase in *Paracoccus denitrificans* is regulated by oxygen and nitric oxide through FnrP and NNR. Microbiology (doi:10.1099/mic.0.054148-0) 10.1099/mic.0.054148-0 PMC3541799 22174385

[37] Bergaust L. Mao Y. Bakken L. R. Frostegård Å. 2010 Denitrification response patterns during the transition to anoxic aespiration and posttranscriptional effects of suboptimal pH on nitrogen oxide reductase in *Paracoccus denitrificans*. Appl. Environ. Microbiol. 76, 6387–6396 10.1128/AEM.00608-10 (doi:10.1128/AEM.00608-10) 20709842 PMC2950438

[38] Bergaust L. Bakken L. R. Frostegard A. 2011 Denitrification regulatory phenotype, a new term for the characterization of denitrifying bacteria. Biochem. Soc Trans. 39, 207–212 10.1042/BST0390207 (doi:10.1042/BST0390207) 21265774

[39] Bergaust L. Shapleigh J. Frostegård Å. Bakken L. R. 2008 Transcription and activities of NOx reductases in *Agrobacterium tumefaciens*: the influence of nitrite, nitrate and oxygen availability. Environ. Microbiol. 10, 3070–3081 10.1111/j.1462-2920.2007.01557.x (doi:10.1111/j.1462-2920.2007.01557.x) 18312398

[40] Liu B. Mao Y. Bakken L. R. Frostegård Å. In preparation. A comparative analysis of genotypes and denitrification phenotypes of strains within the genus *Thauera*.

[41] Mao Y. Bakken L. R. Zhao L. Frostegård A. 2008 Functional robustness and gene pools of a wastewater nitrification reactor: comparison of dispersed and intact biofilms when stressed by low oxygen and low pH. FEMS Microbiol. Ecol. 66, 167–180 10.1111/j.1574-6941.2008.00532.x (doi:10.1111/j.1574-6941.2008.00532.x) 18616585

[42] Yu R. Kampschreur M. J. van Loosdrecht M. C. M. Chandran K. 2010 Mechanisms and specific directionality of autotrophic nitrous oxide and nitric oxide generation during transient anoxia. Environ. Sci. Technol. 44, 1313–1319 10.1021/es902794a (doi:10.1021/es902794a) 20104886

[43] Schreiber F. Loeffler B. Polerecky L. Kuypers M. M. M. de Beer D. 2009 Mechanisms of transient nitric oxide and nitrous oxide production in a complex biofilm. ISME J. 3, 1301–1313 10.1038/ismej.2009.55 (doi:10.1038/ismej.2009.55) 19516281

[44] Kester R .A. deBoer W. Laanbroek H. J. 1997 Production of NO and N_2_O by pure cultures of nitrifying and denitrifying bacteria during changes in aereation. Appl. Environ. Microbiol. 63, 3872–3877 16535707 10.1128/aem.63.10.3872-3877.1997 PMC1389263

[45] Miyahara M. 2010 Potential of aerobic denitrification by *Pseudomonas stutzeri* TR2 to reduce nitrous oxide emissions from waste water treatment plants. Appl. Environ. Microbiol. 76, 4619–4625 10.1128/AEM.01983-09 (doi:10.1128/AEM.01983-09) 20495048 PMC2901746

[46] Baumann B. Snozzi M. VanderMeer J. R. Zehnder A. J. B. 1997 Development of stable denitrifying cultures during repeated aerobic–anaerobic transient periods. Water Res. 31, 1947–1954 10.1016/S0043-1354(97)00053-5 (doi:10.1016/S0043-1354(97)00053-5)

[47] Mahne I. Tiedje J. M. 1995 Criteria and methodology for identifying respiratory denitrifiers. Appl. Environ. Microbiol. 61, 1110–1115 10.1128/aem.61.3.1110-1115.1995 PMC1388392 16534960

[48] Otte S. Grobben N. G. Robertson L. A. Jetten M. S. M. Kuenen J. G. 1996 Nitrous oxide production by *Alcaligenes faecalis* under transient and dynamic aerobic and anaerobic conditions. Appl. Environ. Microbiol. 62, 2421–2426 8779582 10.1128/aem.62.7.2421-2426.1996 PMC168025

[49] McKenney D. J. Drury C. F. Findlay W. I. Mutus B. McDonnell T. Gajda C. 1994 Kinetics of denitrification by pseudomonas fluorescence—oxygen effects. Soil Biol. Biochem. 26, 901–908 10.1016/0038-0717(94)90306-9 (doi:10.1016/0038-0717(94)90306-9)

[50] Kornaros M. Lyberatos G. 1998 Kinetic modelling of *Pseudomonas denitrificans* growth and denitrification under aerobic, anoxic and transient operating conditions. Water Res. 32, 1912–1922 10.1016/S0043-1354(97)00403-X (doi:10.1016/S0043-1354(97)00403-X) 12598188

[51] Remde A. Conrad R. 1991 Metabolism of nitric oxide in soil and denitrifying bacteria. FEMS Microbiol. Ecol. 85, 81–93 10.1111/j.1574-6968.1991.tb04700.x (doi:10.1111/j.1574-6968.1991.tb04700.x)

[52] Cheneby D. Perrez S. Devroe C. Hallet S. Couton Y. Bizouard F. Iuretig G. Germon J. C. Philippot L. 2004 Denitrifying bacteria in bulk and maize-rhizospheric soil: diversity and N2O reducing abilities. Can. J. Microbiol. 50, 469–474 15381970 10.1139/w04-037

[53] Conrad R. 1996 Soil microorganisms as controllers of atmospheric trace gases (H_2_, CO, CH_4_, OCS, N_2_O and NO). Microbiol. Rev. 60, 609–640 8987358 10.1128/mr.60.4.609-640.1996 PMC239458

[54] Kanako T. Ishii S. Nishizawa T. Otsuka S. Senoo K. 2011 Phylogenetic and functional diversity of denitrifying bacteria isolated from various Rice Paddy and Rice-Soybean Rotating fields. Microbes Environ. 26, 30–35 10.1264/jsme2.ME10167 (doi:10.1264/jsme2.ME10167) 21487200

[55] Ma W. K. Bedard-Haughn A. Siciliano S. D. Farrel R. E. 2008 Relationship between nitrifier and denitrifier community composition and abundance in predicting nitrous oxide composition from ephemeral wetland soils. Soil Biol. Biochem. 40, 1114–1123 10.1016/j.soilbio.2007.12.004 (doi:10.1016/j.soilbio.2007.12.004)

[56] Balser T. C. Firestone M. F. K. 2005 Linking microbial community composition and soil processes in a California annual grassland and mixed-conifer forest. Biogeochemistry 73, 395–415 10.1007/s10533-004-0372-y (doi:10.1007/s10533-004-0372-y)

[57] Rich J. J. Myrold D. D. 2004 Community composition and activities of denitrifying bacteria from adjacent agricultural soil, riparian soil and creek sediment in Oregon, USA. Soil. Biol. Biochem. 36, 1431–1441 10.1016/j.soilbio.2004.03.008 (doi:10.1016/j.soilbio.2004.03.008)

[58] Braker G. Dörsch P. Bakken L. R. 2012 Genetic characterization of denitrifier communities with contrasting intrinsic functional traits. FEMS Mirobiol. Ecol. 79, 542–554 10.1111/j.1574-6941.2011.01237.x (doi:10.1111/j.1574-6941.2011.01237.x) 22092293

[59] Firestone M. K. Firestone R. B. Tiedje J. M. 1980 Nitrous oxide from soil denitrification: factors controlling its biological production. Science 208, 749–751 10.1126/science.208.4445.749 (doi:10.1126/science.208.4445.749) 17771133

[60] Simek M. Cooper J. E. 2002 The influence of soil pH on denitrification: progress towards the understanding of this interaction over the last 50 years. Eur. J. Soil Sci. 53, 345–354 10.1046/j.1365-2389.2002.00461.x (doi:10.1046/j.1365-2389.2002.00461.x)

[61] Baggs E. M. Smales C. M. Bateman E. J. 2010 Changing pH shifts the microbial source as well as the magnitude of N2O emission. Biol. Fertil. Soils 46, 793–805 10.1007/s00374-010-0484-6 (doi:10.1007/s00374-010-0484-6)

[62] Liu B. Mørkved P. T. Frostegård Å. Bakken L. R. 2010 Denitrification gene pools, transcription and kinetics of NO, N_2_O and N_2_ production as affected by soil pH. FEMS Microbiol. Ecol. 72, 407–417 10.1111/j.1574-6941.2010.00856.x (doi:10.1111/j.1574-6941.2010.00856.x) 20370831

[63] Holtan-Hartwik L. Dörsch P. Bakken L. R. 2000 Comparison of denitrifying communities in organic soils: kinetics of NO_3_^−^ and N_2_O reduction. Soil Biol. Biochem. 32, 833–843 10.1016/S0038-0717(99)00213-8 (doi:10.1016/S0038-0717(99)00213-8)

[64] Guo J. H. 2010 Significant acidification in major Chinese croplands. Science 327, 1008–1010 10.1126/science.1182570 (doi:10.1126/science.1182570) 20150447

